# Calycosin Alleviates Sepsis-Induced Acute Lung Injury *via* the Inhibition of Mitochondrial ROS-Mediated Inflammasome Activation

**DOI:** 10.3389/fphar.2021.690549

**Published:** 2021-10-19

**Authors:** Yu Xia, Yuanbao Cao, Yao Sun, Xiuying Hong, Yingyan Tang, Juan Yu, Hongjuan Hu, Wenjia Ma, Kailun Qin, Rui Bao

**Affiliations:** ^1^ Department of Pediatrics, LiShui People Hospital, Nanjing, China; ^2^ Department of Clinical Laboratory, LiShui People Hospital, Nanjing, China; ^3^ Department Science and Education, LiShui People Hospital, Nanjing, China; ^4^ Department of Pharmaceutics, School of Pharmacy of Jiangsu University, Zhenjiang, China

**Keywords:** calycosin, sepsis, acute lung injury, inflammasome, reactive oxygen species

## Abstract

Sepsis-induced acute lung injury (ALI) culminates in multiple organ failure via uncontrolled inflammatory responses and requires effective treatment. Herein, we aimed to investigate the effect of calycosin (CA), a natural isoflavonoid, on sepsis-induced ALI. CA attenuated lipopolysaccharide (LPS) and cecal ligation and puncture (CLP)-induced structural damage and inflammatory cell infiltration in lung tissues by histopathological analysis. CA significantly reduced lung wet/dry ratio, inflammatory cell infiltration in bronchoalveolar lavage fluid, and myeloperoxidase activity. Moreover, CA improved the survival of septic mice. CA also substantially inhibited interleukin (IL)-1β and IL-18 levels and cleaved caspase 1 expression and activity in lung tissues. Additionally, CA markedly suppressed oxidative stress by increasing levels of superoxide dismutase and glutathione while decreasing malondialdehyde. *In vitro* assay showed that CA significantly inhibited LPS-induced IL-1β and IL-18 levels and cleaved caspase 1 expression and activity in BMDMs. Moreover, CA blocked the interaction among NLRP3, ASC, and caspase 1 in LPS-treated cells. CA markedly reduced mitochondrial ROS levels. Significantly, compared with CA treatment, the combination of CA and MitoTEMPO (mitochondria-targeted antioxidant) did not further reduce the IL-1β and IL-18 levels and cleaved caspase 1 expression and activity and decreased mitochondrial ROS levels. Collectively, the inhibition of mitochondrial ROS-mediated NLRP3 inflammasome activation contributes to the protective effects of CA, which may be considered a potential therapeutic agent for septic ALI.

## Introduction

Sepsis remains a major problem for human health worldwide. The third international consensus definition for sepsis and septic shock defined sepsis as a “life-threatening dysfunction of organ induced by dysregulation of host response to infection” (Singer et al., 2016). Roughly 1 million Americans have been reported to suffer from sepsis annually, with about a 40% mortality rate (Aziz et al., 2013; Vincent et al., 2014). The cause of septic-induced pathophysiological abnormalities involves the unrestrained reaction of the immune system (“cytokine storm”) to micro-organisms and their products (Hotchkiss and Karl, 2003).

Acute lung injury (ALI) and its severe form, acute respiratory distress syndrome (ARDS), are major health conditions that aggravate the mortality and morbidity of sepsis globally (Aziz et al., 2018). The susceptibility of lungs to injury manifests during sepsis, with over 50% of septic patients developing ALI or ARDS (Sevransky et al., 2009; Gu et al., 2014). Pathologically, the ALI-induced changes are protein-rich fluids accruement in the alveolar cavities, increased permeability of the alveolocapillary membrane, and inflammatory chemokine production for the influx of neutrophils from blood (Wang et al., 2019). Another work has shown that the pathogenesis of septic ALI involves inflammatory cells activation, with macrophages playing vital roles in immune response *via* cytokines and chemokines release (Lomas-Neira et al., 2006). As part of the innate immune system, inflammasomes are large cytosolic multiprotein complexes that are assembled in response to stimuli such as infection and stress with the capability of regulating caspase 1 activation, which mediates inflammatory responses (de Zoete et al., 2014). On the other hand, available evidence has indicated that inflammation without pathogens (sterile inflammation) is mediated through inflammasomes (Ding et al., 2016). The activation of the best characterized nucleotide-binding oligomerization domain-like receptor protein 3 (NLRP3) inflammasome is *via* cellular stress, which may trigger the innate immune defense system. In recent times, the activation of NLRP3 inflammasome has been implicated in the pathogenesis of various diseases. Putatively, several underlying mechanisms of NLRP3 inflammasome activation have been established with an increased production of reactive oxygen species (ROS) widely accepted as a general sensor for alterations in oxidative stress in cells (Tschopp and Schroder, 2010; Abais et al., 2013). Overproduction of ROS has been shown to cause inflammation and damage to the architecture of lung tissues (Blanco-Ayala et al., 2014). Existing literature has posited that paraquat-induced secretion of cytokines (interleukin-IL-1β and IL-18) coupled with activation of NLRP3 inflammasome was evident in macrophages and ALI animal model (Liu et al., 2015). Besides, aldosterone-induced renal tubular injury through activation of NLRP3 inflammasome was mediated by mitochondrial ROS (Ding et al., 2016). Some treatment options for sepsis-induced ALI have been explored, albeit the unmet need to identify safer and more effective drugs that may target mitochondrial ROS-mediated activation of NLRP3 inflammasome.

Although numerous clinical trials have been undertaken, there is no effective drug to provide outstanding outcomes in preventing sepsis-induced ALI (Chen T. et al., 2020). Drugs including dexamethasone, ulinastatin, prednisone, and prednisolone used for clinical treatment of ALI may result in patients with more severe gastrointestinal irritation, allergy, and other side effects (He et al., 2021). Natural products are one of the main sources of drugs or important lead compounds for the treatment of ALI, with the characteristics of few side effects and low toxicity, strong therapeutic efficacy, and multiple targets (Ding et al., 2020). As a well-known immunomodulatory Chinese herbal medicine, Radix Astragali (RA, also called astragalus and Huangqi in Chinese) is developed from the dried root of *Astragalus membranaceus* (Chen Z. et al., 2020). It has been widely explored in the traditional Chinese medicine (TCM) practice for the treatment of various inflammation-induced diseases for decades (Auyeung et al., 2016). Astragalus membranaceus comprises various constituents such as calycosin (CA), polysaccharides, other flavonoids, saponins, and astragalosides (Gao et al., 2014). However, CA is the most potent component in the dry root of RA. Several works have enumerated the health-promoting effects of CA. Among these, diabetes-induced renal inflammation was ameliorated by CA *via* downregulation of IκBα and nuclear factor-κB (NF-κB) p65 phosphorylation (Zhang et al., 2019). Also, CA was reported to attenuate inflammation in 1-methyl-4-phenyl-1,2,3,6-tetrahydropyridine (MPTP)-induced Parkinson’s disease mice through inhibition of the toll-like receptor (TLR)/NF-κB and MAPK pathways (Yang J. et al., 2019). In another work, oxidative stress and inflammatory response in cerulein-induced acute pancreatitis were inhibited by CA through NF-κB and p38 MAPK signaling pathways (Ma et al., 2018). Despite these promising pharmacological activities of CA, its effect on sepsis-induced ALI has not been fully investigated.

In the present study, we examined the protective role of CA in sepsis-induced ALI *in vitro* and *in vivo*. Putatively, it was proposed that CA may ameliorate lipopolysaccharide (LPS) and cecal ligation and puncture (CLP)-induced lung inflammation *via* the inhibition of mitochondrial ROS-mediated inflammasome activation.

## Materials and Method

### Animal and Approval

Animal experiments in this study were done in the Laboratory Animal Research Center of Jiangsu University. The entire animal research protocols for the experiment were performed based on the guidelines issued by the Institutional Animal Care and Use Committees of Jiangsu University. The ethical approval number is UJS-IACUC-2019042101. Male C57BL/6 mice (6 to 8 weeks old) were purchased from Charles River Laboratories (Beijing, China) and housed in polypropylene cages at 21 ± 2°C under a 12 h light/dark cycle and with food and water provided *ad libitum*.

### Murine Model and Treatment

The sepsis-induced ALI model was established by CLP as described in a previous study with mild modification (Rittirsch et al., 2009). Briefly, mice were anesthetized with ketamine (100 mg per kg body weight i. p.) and xylazine (10 mg per kg body weight i. p.) (Rittirsch et al., 2009). A middle abdominal incision was performed and the cecum was exposed, ligated, and punctured through and through with a 20-gauge needle. The ligated cecum was then returned to the peritoneal cavity and the abdomen was closed in two layers. In the sham group, mice underwent exactly the same procedures without performing the ligation and puncture process. CA was purchased from Sigma-Aldrich Corp (St. Louis, MO, #B9938). Mice were divided into five groups (*n* = 10): 1) Sham, 2) CLP, 3) CLP+12.5 mg kg-1 CA, 4) CLP+25 mg kg-1 CA, and 5) CLP+50 mg kg-1 CA. The ligated mice were treated with CA by gavage at 0 and 6 h after CLP. Mice were sacrificed (after 12 h of CLP administration) and the lung tissues were harvested for further analysis. For the survival rate experiment, the ligated mice were treated with CA by gavage once per day. The mortality of mice was monitored every 24 h for 5 days after CLP in each group.

Moreover, sepsis-induced ALI was established by lipopolysaccharide (LPS, Sigma-Aldrich Corp, St. Louis, MO, #L2630) administration followed by application of oleic acid (OA) (Vettorazzi et al., 2015). OA was prepared as a 4% solution in 0.1% bovine serum albumin (BSA). Mice divided into five groups (*n* = 10) as previously described were injected intraperitoneally (i.p.) with 10 mg kg-1 LPS (Sigma Chemical Co., St. Louis, MO, L2880) and 30 min later intravenously (i.v.) with 2.6 μL g-1 oleic acid (OA, Sigma Chemical Co., St. Louis, MO, #O1008). Mice were treated with CA by gavage at 0 and 6 h after LPS administration. After LPS administration for 12 h, mice were sacrificed and lung tissues were harvested for further analysis. For the survival rate experiment, the 20 mg kg-1 LPS-injected mice were treated with CA by gavage once per day. The mortality of mice was monitored every 24 h for 5 days after LPS injection in each group. The CLP or LPS-injected mice for survival study were euthanized when they met moribund criteria for humane endpoints, including hunched posture, dyspnea, and absence of righting reflexes after being laid in a lateral recumbent position.

### Histology and Immunohistochemistry

Lung tissues from mice were fixed with 10% formalin overnight and embedded in paraffin wax. Paraffin blocks were then cut into 5 μm slices and stained with hematoxylin and eosin (H&E). An overall histopathological score was assigned as described previously (Sweeney et al., 2011): 0 = no lesion, 1 = minimal lesion (<10% of the area involved), 2 = mild lesion (10–30% area involved), 3 = moderate lesion (30–50% area involved), 4 = marked lesion (50–80% area involved), and 5 = severe lesion (>80% area involved). For F4/80 (Cell Signaling Technology, Danvers, MA, #70076) immunohistochemistry, lung tissue slides were fixed and stained using a SignalStain^®^ DAB substrate kit (#8059) according to the manufacturer’s instructions.

### Lung Wet to Dry Weight Ratio Analysis

The lower part of the right lung was excised and weighed to obtain the wet weight. Then, the wet lung was dried in an oven at 72°C for 48 h to obtain the dry weight. Lung wet to dry (W/D) weight ratio was calculated to assess the lung edema.

### Analysis of Bronchoalveolar Lavage Fluid

Briefly, mice were sacrificed and the tracheae were exposed. Ice-PBS (1 ml) was injected into the lungs and extracted with a syringe. This step was repeated three times. The collected BALF was centrifuged at 1,000 g for 5 min at 4°C. The supernatant was used to detect the levels of interleukin (IL)-1β and IL-18 by ELISA assay. The cell pellet was suspended in 1 ml, and the total number of cells was determined with a hemocytometer. Then, the cells were subjected to Wright–Giemsa staining according to the manufacturer’s instruction. The counts of different cells, including macrophages, lymphocytes, and neutrophils, were viewed and determined with a bright-field microscope.

### Measurement of Biochemical Markers in Lung Tissue

In brief, lung tissue was homogenized in ice-PBS and the supernatants were harvested after centrifugation. The levels of malondialdehyde (MDA, #A003-1-1), superoxide dismutase (SOD, #A001-1-1), glutathione (GSH, #A006-2-1), and myeloperoxidase (MPO, #A004-1-1) in lung tissues were measured using commercial kits (Nanjing Jiancheng Bioengineering Institute, Nanjing, China) in accordance with the manufacturer’s protocol.

### Bone Marrow-Derived Macrophages Differentiation and Stimulation

Bone marrow cells were obtained by flushing the femurs and tibias from 6–8 weeks old mice and cultured in Dulbecco’s modified Eagle medium containing 50 ng/ml recombinant macrophage-colony stimulating factor (M-CSF, Peprotech, Cranbury, NJ, #315–02), 10% fetal bovine serum (Gibco, Grand Island, NY), penicillin (100 U/mL), and streptomycin (100 μg/ml) under a humidified 5% (v/v) CO_2_ atmosphere at 37°C for 7 days to form macrophages. BMDMs were treated with 100 ng/ml LPS and 5 mM ATP to activate the inflammasomes.

### RNA Extraction and Real-Time PCR

Lung tissue was homogenized in TRIzol^TM^ reagent (Invitrogen, Carlsbad, CA) and total RNA was extracted according to the manufacturer’s instructions. Total RNA from lung tissue was reverse-transcribed using a RETROscript^®^ reverse transcription kit (Invitrogen, Carlsbad, CA, #AM1710). Next, real-time PCR was carried out using cDNA, primers, and SYBR Green Master Mix (Invitrogen, Carlsbad, CA) with an ABI Prism 7500 sequence detection system (Applied Biosystems, Foster City, CA) following the manufacturer’s instructions. Amplification of β-actin was used as an internal control. The primers used are summarized in Table 1.

**TABLE 1:** Primer sequences.

Name	Forward/Reverse	Primer sequence (5′ to 3′)
IL-1β	F	TCC TGT GTA ATG AAA GAC GGC
—	R	ACT CCA CTT TGC TCT TGA CTT C
IL-18	F	CTT CGT TGA CAA AAG ACA GCC
—	R	CAC AGC CAG TCC TCT TAC TTC
β-Actin	F	CTT CGT TGA CAA AAG ACA GCC
—	R	CAC AGC CAG TCC TCT TAC TTC

### ELISA Assay

Cell culture medium and the BALF samples were harvested after the stimulation or surgical procedures. Measurement of IL-1β (#MLB00C) or IL-18 (#7625) concentration was determined by ELISA kits (R&D Systems, Inc., Minneapolis, MN) according to the manufacturer’s instruction.

### Western Blot and Co-Immunoprecipitation Assay

Briefly, the protein was extracted from lung tissue and BMDMs with RIPA lysis buffer containing protease inhibitor cocktail (Cell Signaling Technology, Danvers, MA). The protein concentration was determined by a Pierce™ Bicinchoninic acid (BCA) protein assay kit (Pierce, Rochford, IL, #23227) according to the manufacturer’s instructions. An equal amount of denatured protein was loaded to SDS-PAGE gel, separated, and subsequently electro-transferred onto a polyvinylidene fluoride membrane. The membrane was blocked with 5% non-fat milk, incubated with the primary and the horseradish peroxidase-conjugated secondary antibodies (Cell Signaling Technology, Danvers, MA). Blots were visualized using Chemiluminescent Substrate. ImageJ software was used for densitometric analysis. Densitometric values were normalized to β-actin. Mouse reactive inflammasome antibody sampler kit (#20836) purchased from Cell Signaling Technology (Danvers, MA) was used as primary antibody. For co-immunoprecipitation assay, the lysates were immunoprecipitated with NLRP3 antibody or IgG control antibody with protein A/G beads. After washing the beads, the protein bands were visualized by western blot analysis.

### Flow Cytometric Analysis

To evaluate the intracellular ROS production, the treated cells were harvested and incubated with 10 μM 2,7-dichlorofluorescein diacetate (DCFH-DA, Sigma-Aldrich Corp, St. Louis, MO, #D6883) at 37°C for 20 min during darkness prior to washing with ice-PBS twice. The DCF fluorescence distribution of 1 × 10^4^ events was detected by a FACScan flow cytometry at an excitation wavelength of 488 nm and an emission wavelength of 525 nm. For dihydroethidium (DHE) staining, the treated cells were harvested and incubated with 5 μM DHE (Beyotime Biotechnology, Shanghai, China, S0063) at 37°C for 30 min during darkness prior to washing with ice-PBS twice. The DHE fluorescence distribution of 1 × 10^4^ events was detected by a FACScan flow cytometry at an excitation wavelength of 370 nm and an emission wavelength of 610 nm. To evaluate superoxide production by mitochondria, the treated cells were harvested and incubated with a 10 μM MitoSOX^TM^ Red reagent (2.5 μM, Invitrogen, Carlsbad, CA, #M36008) at 37°C for 30 min in the dark before washing with warm buffer twice. The MitoSOX fluorescence distribution of 1 × 10^4^ events was detected by a FACScan flow cytometry at an excitation wavelength of 488 nm and an emission wavelength of 620 nm. Fluorescent signal intensity was analyzed with the percentage of subpopulations by FlowJo software.

### Caspase 1 Activity Assay

Caspase 1 activity was measured by a colorimetric assay according to the manufacturer’s instruction (Beyotime Biotechnology, Shanghai, China, #C1101). Briefly, the protein was extracted from lung tissues and BMDMs with lysis buffer. The protein concentration was determined by a BCA protein assay kit (Pierce, Rochford, IL, #23227). Protein (100 μg) was incubated with the substrate of caspase 1 (Ac-YVAD-pNA, 10 μL, 2 mmol/L) at 37°C for 2 h. The absorbance values of pNA at 405 nm were determined by a spectrophotometer. A standard curve of pNA was performed to calculate the caspase 1 activity.

### Statistical Analysis

Means ± standard error of the mean (SEM) was used to express the data. Statistical analysis was performed by one-way analysis of variance (ANOVA) followed by Dunnett’s multiple comparison test using GraphPad Prism 7 software. The Kaplan–Meier method was used to compare the mortality differences among groups. A *p* < 0.05 was considered statistically significant.

## Results

### Calycosin Protects Against Sepsis-Induced ALI

**FIGURE 1:**
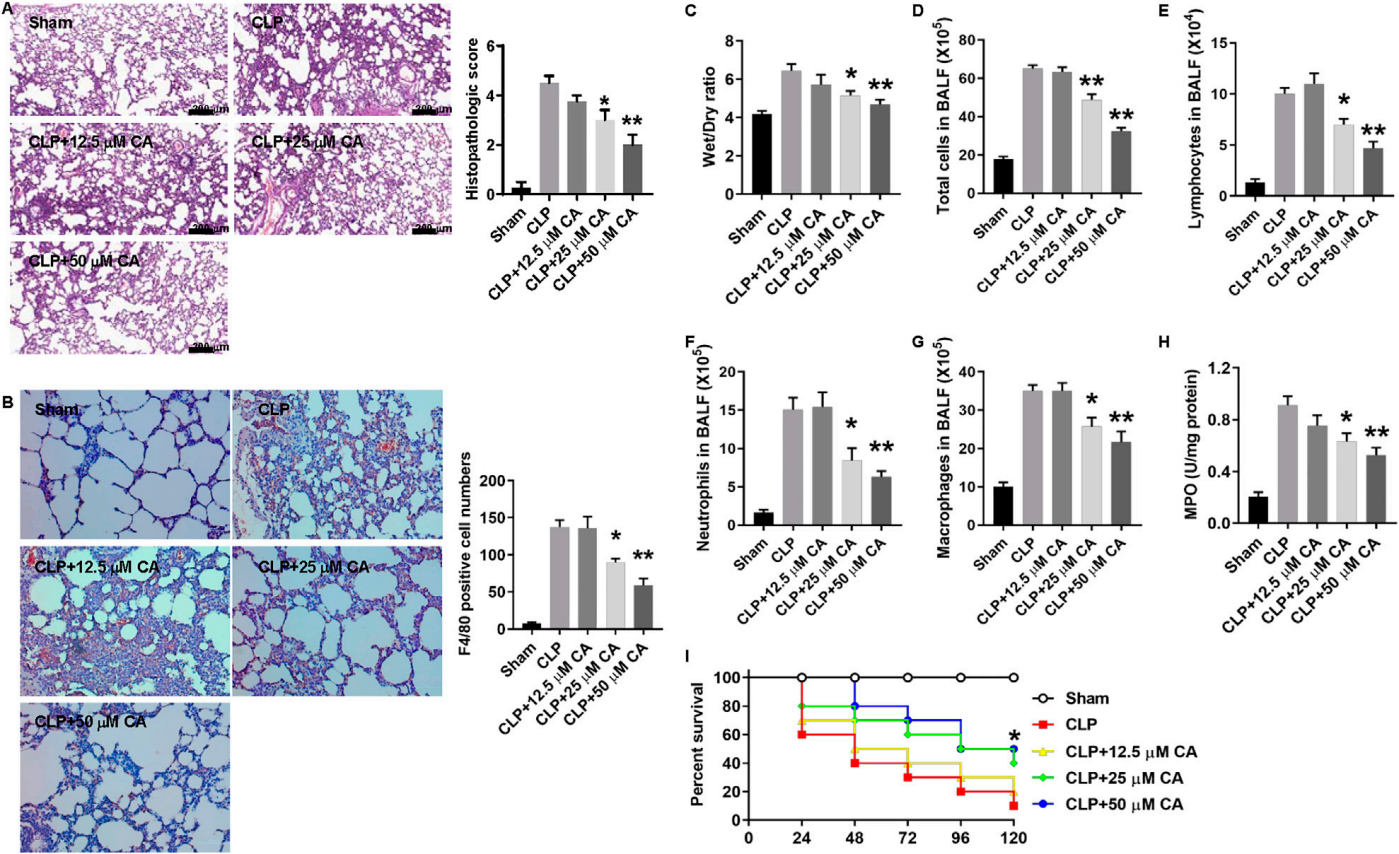
Calycosin protects against cecal ligation and puncture (CLP)-induced septic acute lung injury. Mice were anesthetized and CLP procedure was performed. Sham mice were operated in the same procedure as CLP mice except for the ligation and puncture. The ligated mice were treated with calycosin by gavage at 0 and 6 h after CLP. After CLP for 12 h, mice were anesthetized and sacrificed. **(A)** Lung tissues were stained with hematoxylin and eosin. Histopathologic scores were calculated according to the severity of lung damage. **(B)** Lung tissue slides were stained by F4/80 immunohistochemistry. The numbers of positive cells were counted and statistically analyzed. **(C)** The lower part of the right lung was excised and weighed to obtain the wet weight. Then, the wet lung was dried in an oven at 72°C for 48 h to obtain the dry weight. The ratio of wet to dry (W/D) weight was calculated to assess the lung edema. **(D)** The total cell number in the BALF was determined. **(E–G)** The cell numbers in the BALF, including macrophages, lymphocytes, and neutrophils, were determined by the Wright–Giemsa staining. **(H)** MPO activity was determined. All the quantitative data **(A–G)** are presented as means ± SEM. *p < 0.05; **p < 0.01 vs. the CLP group. **(I)** Mice were treated with different doses of calycosin by gavage once per day after CLP. The mortality of mice was monitored every 24 h for 5 days after CLP (10 mice per group). The Kaplan–Meier method was used to compare the differences among groups. *p < 0.05 vs. the CLP group.

To evaluate the potential protective effects of CA against sepsis-induced ALI, we used two different murine sepsis models. In the CLP or LPS/OA-injection model, mice were treated with different doses of CA at 0 and 6 h. Within 12 h, none of the mice died in these two models. After CLP or LPS injection for 12 h, lung tissues from mice were collected and H&E staining was used to evaluate the damage of the lung. As shown in Figure 1A, the ligated mice have a heavy infiltration of inflammatory cells, exudative changes in the peribronchial layer of the bronchi, edema, and fibrin deposition, while they are attenuated by CA treatment (*p* < 0.05, CLP+20; *p* < 0.01, CLP+50) and the histopathologic score was significantly decreased. Moreover, F4/80 immunohistochemistry results showed that CA treatment inhibited macrophage infiltration in lung tissue. The number of F4/80 positive cells was significantly decreased in CA-treated mice compared with the no-treatment group (Figure 1B). The W/D ratio of lung tissues, which signifies pulmonary edema, was significantly decreased in CA-treated groups compared to the CLP group (Figure 1C). Total cell numbers including macrophages, lymphocytes, and neutrophils and MPO activity markedly decreased in CA-treated groups compared with the CLP group (Figures 1E–H). Interestingly, the survival rate of mice in the CA-treated groups was significantly improved compared with the CLP group (Figure 1I).

**FIGURE 2:**
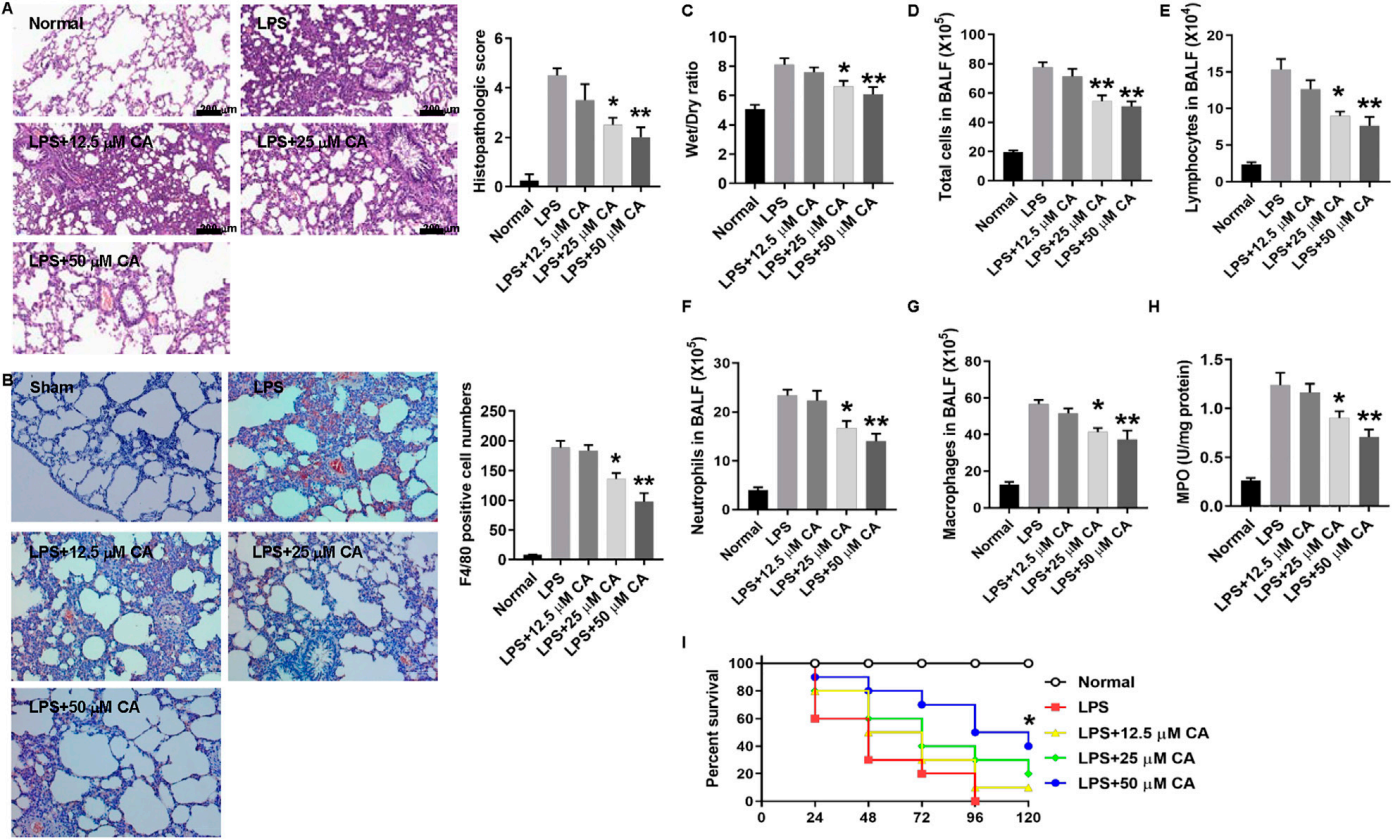
Calycosin protects against lipopolysaccharide (LPS)-induced septic acute lung injury. Mice were injected intraperitoneally (i.p.) with 10 mg kg^−1^ LPS and 30 min later intravenously (i.v.) with 2.6 μLg^−1^ oleic acid (OA). Mice were treated with calycosin by gavage at 0 and 6 h after LPS injection. After LPS injection for 12 h, mice were anesthetized and sacrificed. **(A)** Lung tissues were stained with hematoxylin and eosin. Histopathologic scores were calculated according to the severity of lung damage. **(B)** Lung tissue slides were stained by F4/80 immunohistochemistry. The numbers of positive cells were counted and statistically analyzed. **(C)** The lower part of the right lung was excised and weighed to obtain the wet weight. Then, the wet lung was dried in an oven at 72°C for 48 h to obtain the dry weight. The ratio of wet to dry (W/D) weight was calculated to assess the lung edema. **(D)** The total cell number in the BALF was determined. **(E–G)** The cell numbers in the BALF, including macrophages, lymphocytes, and neutrophils, were determined by the Wright–Giemsa staining. **(H)** MPO activity was determined. All the quantitative data **(A–G)** are presented as means ± SEM. *p < 0.05; **p < 0.01 vs. the LPS group. **(I)** Mice were treated with different doses of calycosin by gavage once per day after LPS injection. The mortality of mice was monitored every 24 h for 5 days after LPS injection (10 mice per group). The Kaplan–Meier method was used to compare the differences among groups. *p < 0.05 vs. the LPS group.

Figure 2 shows the potential beneficial effect of CA on LPS-induced septic ALI. Through H&E staining, it was observed that the sectioned lung tissue of mice in the normal group showed no abnormal changes with normal structural alveolar (Figure 2A, Normal), while that of LPS-treated mice (Figure 2A, LPS) was pathologically altered and diffused *via* the formation of edema in the interstice, apparent infiltration of inflammatory cells, and structural disarray of alveolar and hemorrhage. Nonetheless, CA treatment improved the histopathologic changes with a substantial (*p* < 0.05, LPS+20; *p* < 0.01, LPS+50) decrease in the score compared with the LPS group. Consistent with the results of Figure 1B, the number of F4/80 positive cells was also significantly decreased in CA-treated mice compared with the no-treatment group (Figure 2B). Moreover, the W/D ratio in lung tissue was significantly reduced by CA treatment (*p* < 0.05, LPS+20; *p* < 0.01, LPS+50) compared with the LPS group (Figure 2C). Besides, an obvious (*p* < 0.01) decrease in total cell number in BALF of mice treated with CA was observed compared with that of the LPS group (Figures 2D–G). In addition, CA substantially (*p* < 0.05) improved the survival of the mice after the LPS challenge (Figure 2I). Overall, these results suggest that CA may dose-dependently protect the mice against CLP and LPS-induced septic ALI.

### Calycosin Reduces IL-1β and IL-18 mRNA and Protein Levels in Lung Tissue and BALF

**FIGURE 3:**
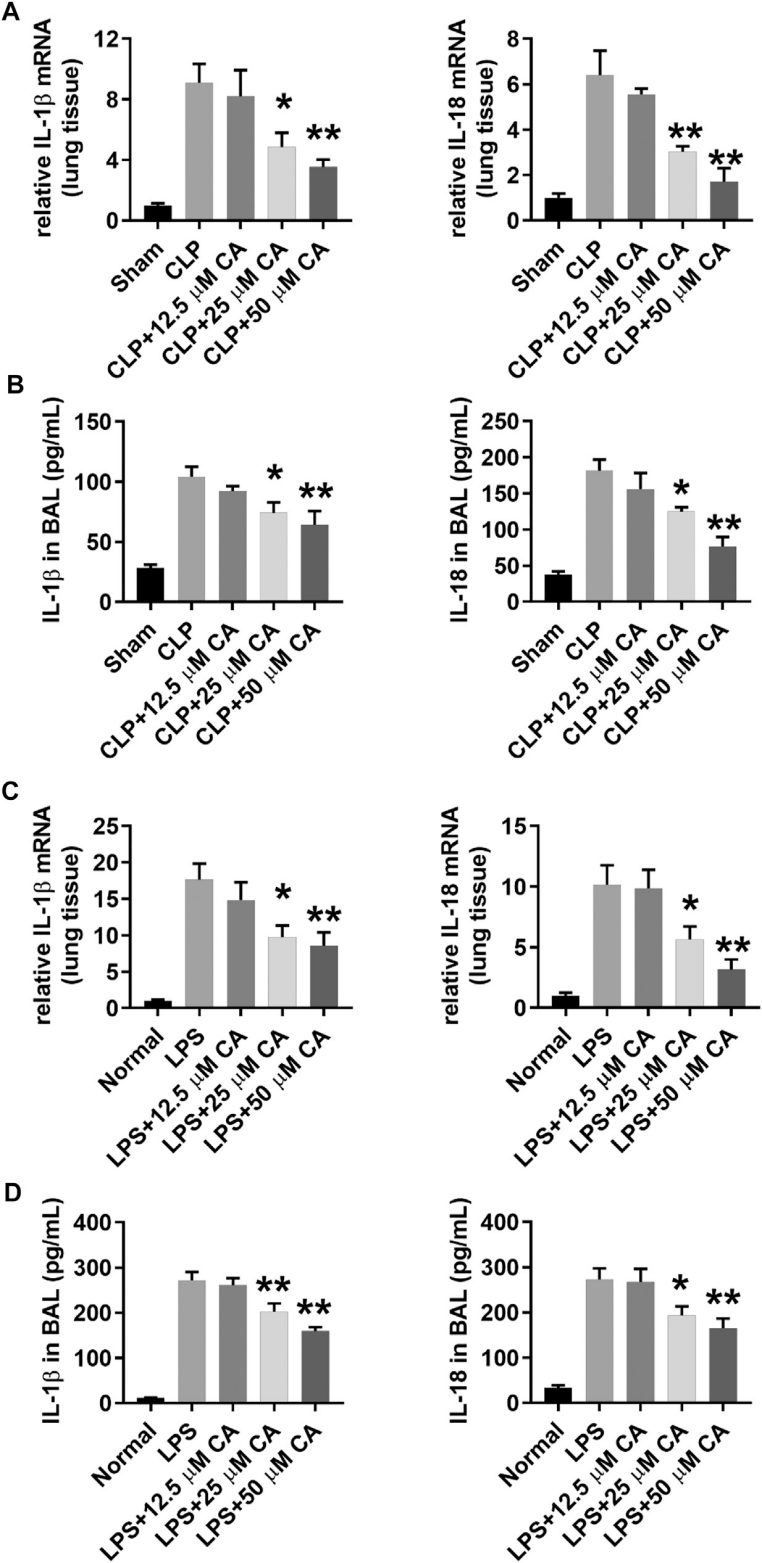
Calycosin reduces IL-1β and IL-18 mRNA and protein levels in lung tissue and BALF. The ligated or LPS-injected mice were treated with calycosin by gavage at 0 and 6 h. After 12 h, mRNA expression and protein levels in lung tissue and BALF were determined by real-time PCR and ELISA assay (10 mice per group). Data are presented as means ± SEM. *p < 0.05; **p < 0.01 vs. the CLP or LPS group.

After establishing the two models, real-time PCR and ELISA assay were used to determine IL-1β and IL-18 mRNA and protein levels in lung tissue and BALF. As depicted in Figure 3, the IL-1β and IL-18 mRNA and protein levels increased after CLP and LPS injection. However, administration of CA significantly reduced the production of IL-1β (*p* < 0.05, 25 mg kg-1; *p* < 0.01, 50 mg kg-1) and IL-18 (*p* < 0.01) mRNA level in lung tissue compared with CLP group (Figure 3A). Likewise, protein levels of IL-1β and IL-18 (*p* < 0.05, 25 mg kg-1; *p* < 0.01, 50 mg kg-1) in the BALF of CA-treated mice decreased obviously compared to those in the CLP group (Figure 3B). In the LPS model, IL-1β and IL-18 levels (*p* < 0.05, 25 mg kg-1; *p* < 0.01, 50 mg kg-1) were substantially reduced in lung tissues of mice treated with CA (Figure 3C). At the protein level, synthesis of IL-1β and IL-18 (*p* < 0.05, 25 and *p* < 0.01, 50 mg kg-1) markedly diminished in BALF of CA-treated mice (Figure 3D). Altogether, CA (in a dose-dependent fashion) showed anti-inflammatory effects *via* reducing IL-1β and IL-18 levels in lung tissue and BALF of CA-treated mice.

### Calycosin Inhibits NLRP3 Inflammasome Activation in Lung Tissue

**FIGURE 4:**
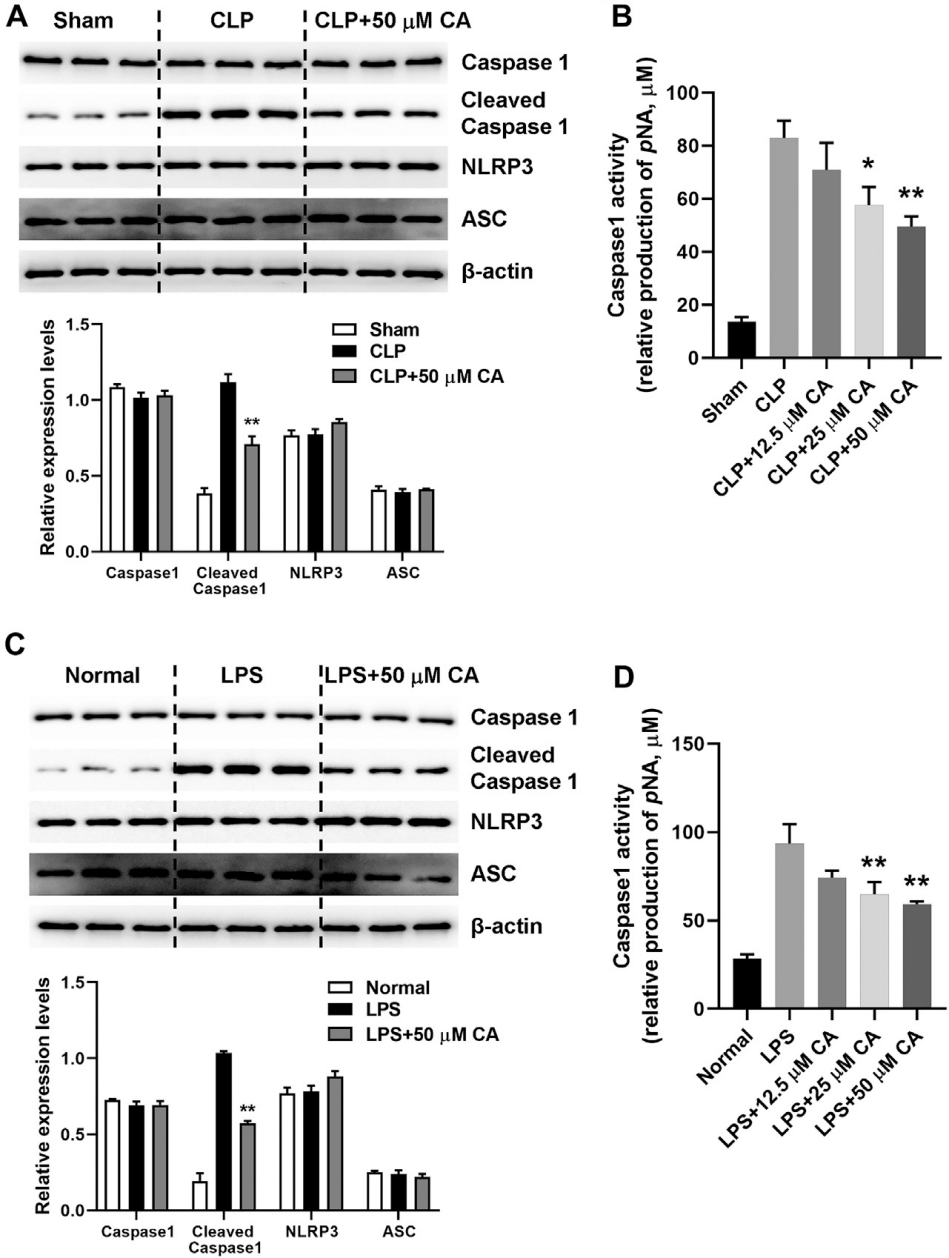
Calycosin inhibits NLRP3 inflammasome activation in lung tissue. The ligated or LPS-injected mice were treated with calycosin by gavage at 0 and 6 h. After 12 h, NLRP3 inflammasome associated protein levels in lung tissue were determined by western blot analysis. The quantitative densitometry of the protein expressions was determined by Image J software. Densitometric values were normalized to β-actin. The caspase 1 activity in lung tissue was determined by a commercial kit. Data are presented as means ± SEM. *p < 0.05; **p < 0.01 vs. the CLP or LPS group.

NLRP3 inflammasome activation triggers caspase 1 activation and IL-1β maturation (Yang Y. et al., 2019). The mechanism of anti-inflammatory effect of CA was evaluated by detecting NLRP3 inflammasome activation in lung tissue of mice with sepsis-induced ALI after 12 h *via* western blot analysis. As displayed in Figure 4, the cleaved caspase 1 protein levels generally increased upon CLP or LPS challenge. However, CLP (Figure 4A) and LPS-induced (Figure 4C) high protein levels of cleaved capase 1 were substantially (*p* < 0.01) suppressed when the mice received CA (50 mg kg-1). Contrarily, caspase 1 activity was significantly inhibited by CA treatment compared with CLP (*p* < 0.05, CLP+25; *p* < 0.01, CLP+50) or LPS (*p* < 0.01) groups (Figures 4B,D). Additionally, as shown in Figures 4A,C, levels of NLRP3 protein did not alter significantly after CA (50 mg kg^−1^) treatment compared with CLP or LPS groups (Figures 4A,C). These data indicated that CA inhibits NLRP3 inflammasome activation in lung tissue.

### Calycosin Suppresses Oxidative Stress in Lung Tissue

**FIGURE 5:**
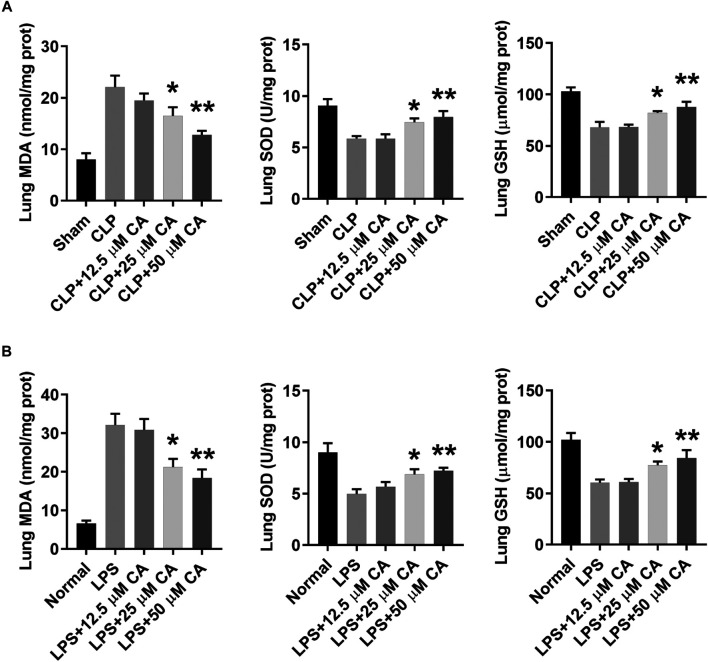
Calycosin inhibits oxidative stress in lung tissue. The ligated or LPS-injected mice were treated with calycosin by gavage at 0 and 6 h. After 12 h, malondialdehyde (MDA), superoxide dismutase (SOD), and glutathione (GSH) levels in lung tissues were determined by commercial kits. Data are presented as means ± SEM. *p < 0.05; **p < 0.01 vs. the CLP or LPS group.

To identify the involvement of oxidative stress in CLP or LPS-induced septic ALI, the levels of MDA, SOD and GSH were determined in lung tissue at 12 h. In general, the levels of MDA in lung tissue of CLP or LPS-induced septic ALI mice markedly increased, while that of SOD and GSH decreased (Figure 5). However, when these mice received CA (*p* < 0.05-CLP+25, *p* < 0.01-CLP+50), the MDA was obviously reduced, while SOD and GSH increased significantly compared with the CLP or LPS group. Together, these results indicate CA may suppress oxidative stress in lung tissue of sepsis-induced ALI mice by increasing levels of SOD and GSH, while reducing MDA concentration.

### Calycosin Inhibits LPS-Induced NLRP3 Inflammasome Activation in BMDMs.

**FIGURE 6:**
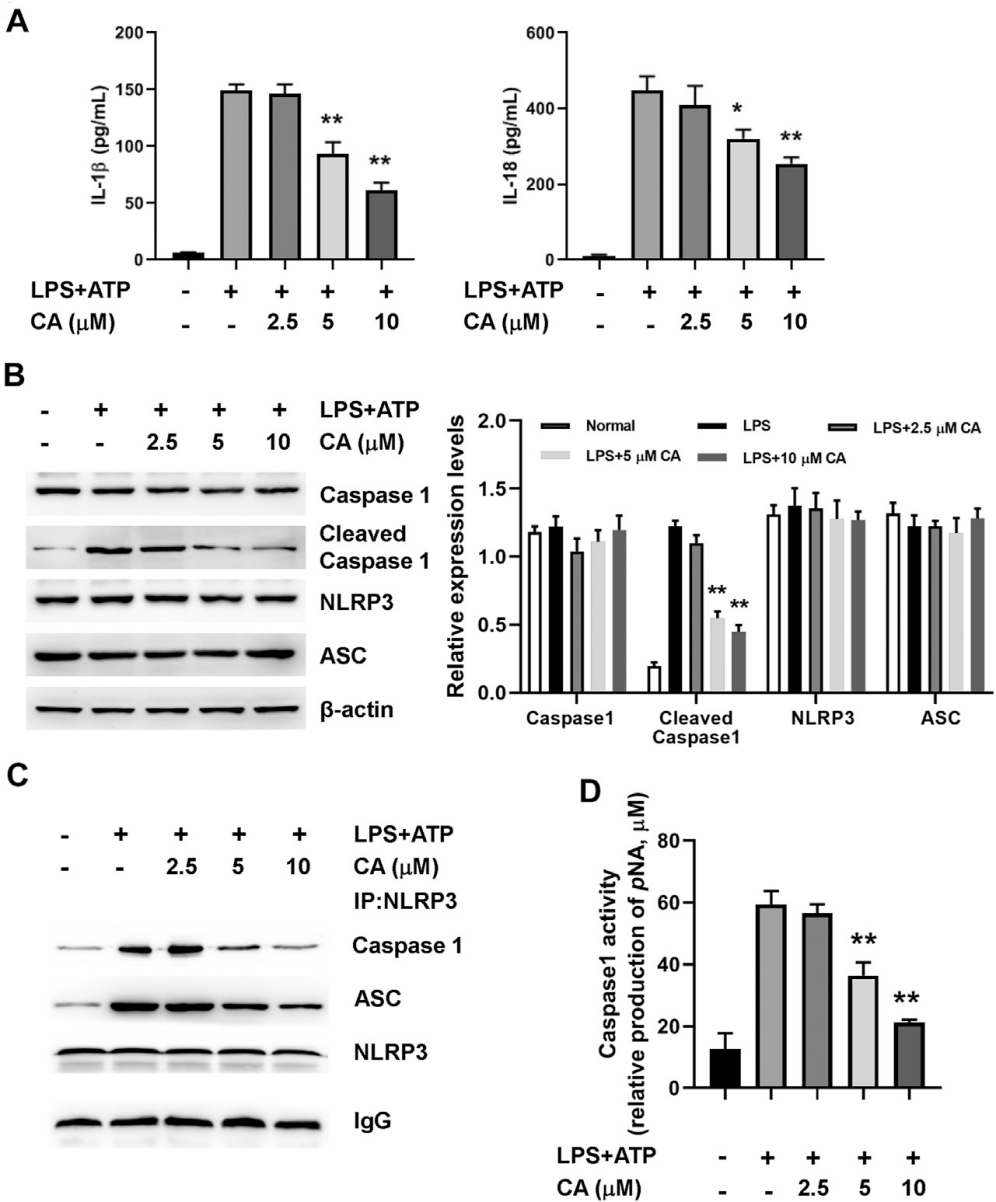
Calycosin inhibits LPS-induced NLRP3 inflammasome activation in BMDMs. BMDMs cells were treated with the different concentrations of calycosin in the presence of 500 ng/ml LPS and 5 mM ATP for 24 h. **(A)** The levels of IL-1β and IL-18 in the supernatant were determined by ELISA assay. **(B)** The protein levels of NRLP3 inflammasome were determined by western blot. The quantitative densitometry of the protein expressions was determined by Image J software. Densitometric values were normalized to β-actin. **(C)** The effect of CA on the interaction among NLRP3, ASC, and caspase 1 were determined by co-immunoprecipitation assay. **(D)** The caspase 1 activity in BMDMs was determined by a commercial kit. Data are presented as means ± SEM. *p < 0.05; **p < 0.01 vs. the LPS+ATP group.

In order to further ascertain the potential mechanism underlying the anti-inflammatory properties of CA, we determined the activation of NLRP3 inflammasome in BMDMs after 24 h of LPS induction. As shown in Figure 6A, the levels of IL-1β and IL-18 increased obviously in BMDMs after 24 h of LPS+ATP treatment. In comparison with the LPS group, CA (5 and 10 µM) treatment could evidently decrease IL-1β (*p* < 0.01) and IL-18 levels (*p* < 0.05, 5 µM; *p* < 0.01, 10 µM) in BMDMs. Increased protein levels of cleaved caspase 1 in the LPS+ATP challenge was markedly diminished in BMDMs after CA treatment compared with the LPS group (*p* < 0.01, Figure 6B). Meanwhile, the protein levels of NLRP3 in BMDMs of CA-treated mice did not significantly differ from the LPS+ATP group. Co-immunoprecipitation assay results showed that CA at 5 and 10 μM significantly inhibited the interaction among ASC, caspase 1, and NLRP3 in BMDMs (Figure 6C). As displayed in Figure 6D, caspase 1 activity was substantially inhibited by CA compared with that in the LPS group (*p* < 0.01). Overall, CA treatment may inhibit NLRP3 inflammasome activation by decreasing IL-1β and IL-18 levels and suppressing caspase 1 activity.

### Calycosin Reduces LPS-Induced Mitochondrial ROS Production in BMDMs

**FIGURE 7:**
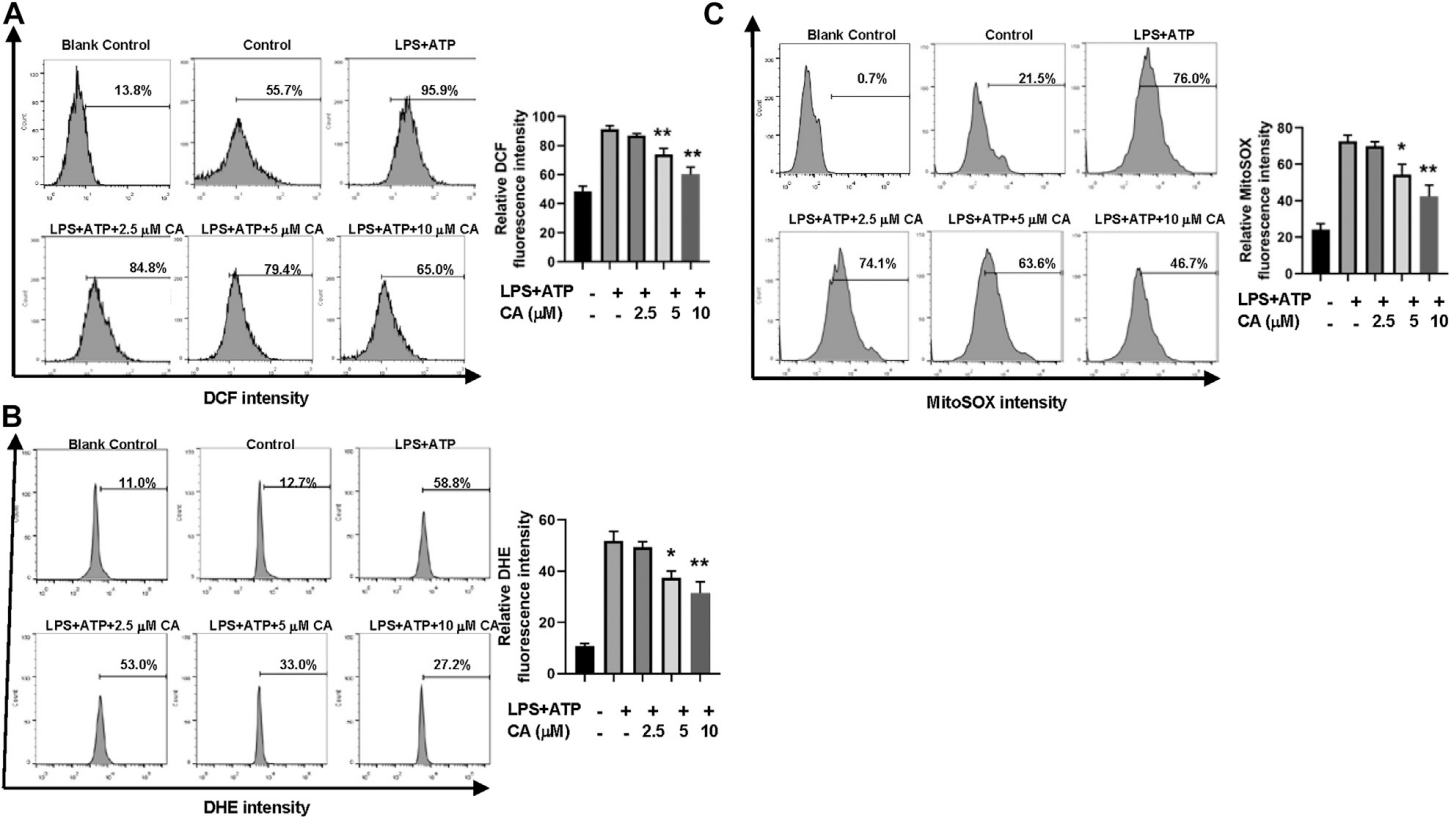
Calycosin reduces LPS-induced mitochondrial ROS production in BMDMs. BMDMs were treated with the different concentrations of calycosin in the presence of 500 ng/ml LPS and 5 mM ATP for 12 h. **(A)** Cells were harvested and further incubated with 2,7-dichlorofluorescein diacetate (DCFH-DA, 10 μM) for 20 min at 37°C in the darkness. Cell samples were acquired with a FACScan flow cytometer with an excitation wavelength of 488 nm and an emission wavelength of 525 nm. **(B)** Cells were harvested and further incubated with 5 μM DHE for 30 min at 37°C in the darkness. Cell samples were acquired with a FACScan flow cytometer with an excitation wavelength of 370 nm and an emission wavelength of 610 nm. **(C)** Cells were harvested and further incubated with MitoSOX^TM^ Red (5 μM) for 10 min at 37°C in the darkness. Cell samples were acquired with a FACScan flow cytometer with an excitation wavelength of 488 nm and an emission wavelength of 620 nm. All fluorescent signal intensities were analyzed by FlowJo software. Data are presented as means ± SEM. *p < 0.05; **p < 0.01 vs. the LPS+ATP group.

Through flow cytometric analysis, the underlying mechanism of CA on inhibition of NLRP3 inflammasome activation was clarified by measuring the production of ROS induced by LPS in BMDMs. In general, the concentration of total ROS in BMDMs increased after LPS stimulation (Figures 7A,B). In contrast, the increased ROS level was evidently decreased after CA treatment in comparison with the LPS group (*p* < 0.01) by DCFH-DA and DHE staining. Next, we detected whether mitochondria were involved in the decrease of CA-induced ROS. MitoSOX^TM^ Red was used to measure the level of mitochondrial ROS in CA-treated BMDMs. Compared to the LPS group (*p* < 0.05, 5 µM; *p* < 0.01, 10 µM), high levels of mitochondrial ROS were reduced substantially after CA treatment (Figure 7C). Our data indicated that CA could reduce mitochondrial ROS production in BMDMs in a dose-dependent manner.

### Calycosin Inhibits LPS-Induced NLRP3 Inflammasome Activation by Reducing Mitochondrial ROS Production.

**FIGURE 8:**
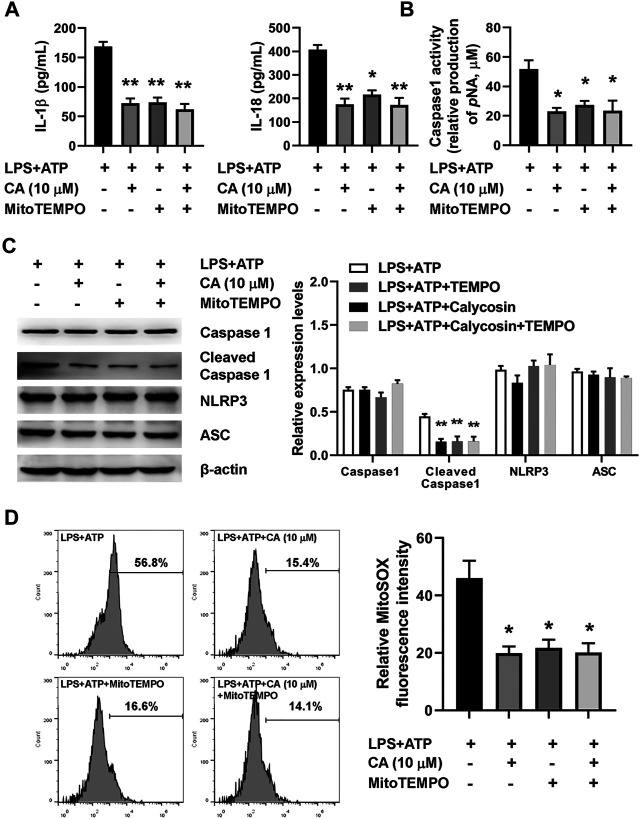
Calycosin inhibits LPS-induced NLRP3 inflammasome activation through reducing mitochondrial ROS production in BMDMs. The LPS+ATP-induced BMDMs were treated with calycosin (10 μM) in the absence or presence of MitoTEMPO (10 μM) for 24 h. **(A)** The levels of IL-1β and IL-18 in the supernatant were determined by ELISA assay. **(B)** The caspase 1 activity in BMDMs was determined by a commercial kit. **(C)** The protein levels of NRLP3 inflammasome were determined by western blot. The quantitative densitometry of the protein expressions was determined by Image J software. Densitometric values were normalized to β-actin. **(D)** The LPS+ATP-induced BMDMs were treated with calycosin (10 μM) in the absence or presence of MitoTEMPO (10 μM) for 12 h. Cells were harvested and further incubated with MitoSOX^TM^ Red (5 μM) for 10 min at 37°C in the darkness. Cell samples were acquired with a FACScan flow cytometer (Becton Dickinson, United States) with an excitation wavelength of 620 nm and an emission wavelength of 580 nm. Fluorescent signal intensity was analyzed by FlowJo software. Data are presented as means ± SEM. *p < 0.05; **p < 0.01 vs. the LPS+ATP group.

To further understand the mechanism underlying NLRP3 inflammasome activation induced by LPS in CA-treated BMDMs, MitoTEMPO, a mitochondria-targeted superoxide dismutase, was used to measure mitochondrial ROS production in BMDMs. The marked increase in the concentration of IL-1β, IL-18, cleaved caspase 1 expression, caspase 1 activity, and mitochondrial ROS was observed after the LPS challenge (Figures 8A–D). Compared with the LPS+ATP group, CA (10 μM) and MitoTEMPO (10 μM) alone treatment prominently decreased levels of IL-1β and IL-18, the activity of caspase 1, cleaved caspase 1 expression, and mitochondrial ROS in BMDMs. Importantly, the results from the combined effect of CA and MitoTEMPO also showed an obvious decrease in BMDM. However, compared with CA alone treatment, levels of IL-1β and IL-18, the activity of caspase 1, cleaved caspase 1 expression, and mitochondrial ROS did not show a further reduction in the combination treatment group, suggesting that CA might inhibit mitochondrial ROS-mediated NLRP3 inflammasome activation induced by LPS.

## Discussion

In this study, we indicated that a crucial protective role of CA in sepsis-induced ALI. For the first time, we demonstrated that CA could diminish ALI induced by sepsis in mice by inhibiting inflammasome activation. A cascade of inflammatory mediators released from neutrophils, macrophages, mast cells, and endothelial cells led to the increase of pulmonary microvascular permeability and interstitial and alveolar pulmonary edema in the development of ALI pathogenesis (Herold et al., 2013). Therefore, finding the candidate and elucidating its underlying molecular mechanisms associated with inflammation may be useful in treating ALI. CA, an isoflavonoid, is a major active component of *Astragalus membranaceus*, which has been widely used in the treatment of inflammatory diseases such as diabetes-induced renal inflammation (Zhang et al., 2019), allergic contact dermatitis (Jia et al., 2018), and myocardial infarction (Cheng et al., 2015). However, few reports have revealed the protective effects of CA on sepsis-induced ALI and its potential mechanism. Here, our results have shown that in CLP or LPS-injected mice, CA attenuated sepsis-induced structural damage and inflammatory cell infiltration in lung tissue and reduced lung wet/dry ratio and inflammatory cell infiltration in BALF and MPO activity. Moreover, CA improved the survival of septic mice induced by CLP or LPS. It has been reported that CA rebalances insulin sensitivity and inflammatory response in gestational diabetes mellitus by suppressing ring finger protein 38 (RNF38) expression (Li et al., 2020). CA not only inhibited the toll-like receptor 4-related protein expression but also reduced NF-κB activation atopic dermatitis model (Tao et al., 2017). Our results from CLP or LPS-injected mice showed that CA reduced IL-1β and IL-18 levels, which indicated that inflammasome activation was inhibited by CA. In line with the ELISA assay, Figure 4 also shows that CA significantly reduced cleaved caspase 1 expression and activity in lung tissues. Although many studies have reported the multiple functions of CA in inflammatory diseases, the effects of CA on inflammasome activation remain unclear. So far, only one study has reported that CA reduced the levels of sirtuin 1 (Sirt1)-NLRP3 and related proteins in doxorubicin-treated cells and mice hearts, suggesting that CA offers the possibility for the treatment of cardiotoxicity through inhibition of inflammasome (Zhai et al., 2020). Our study provides a rationale for CA to inhibit inflammasome activation in the treatment of ALI.

Inflammasomes, including NLRP3 and ASC, assemble and release inflammatory cytokines (e.g., IL-1β and IL-18) in response to tissue damage after pathogen-associated molecular patterns (PAMPs) or danger-associated molecular patterns (DAMPs) were recognized by pattern recognition receptors (PRRs) (Lamkanfi and Dixit, 2014). Herein, we found that CA significantly decreased cleaved caspase 1 expression but not NLRP3 and ASC. However, our result showed that CA significantly inhibited the interaction among ASC, caspase 1, and NLRP3 in BMDMs. This result indicates that CA inhibits NLRP3 inflammasome activation by blocking the formation of the NLRP3 inflammasome complex, which may contribute to the inhibition of caspase 1 activity. It was known that LPS and ATP are well-characterized stimulators to trigger inflammasome complex formation (Bednash and Mallampalli, 2016). Our *in vitro* results showed that CA inhibited LPS-induced NLRP3 inflammasome activation in BMDMs and decreased IL-1β and IL-18 levels, caspase 1 activity, and cleaved caspase 1 expression. The mechanisms of inflammasome activation are sophisticated as they respond to injury and danger. It has been reported that caspase 11 is required for the secretion of IL-1β and IL-18 in macrophages after stimulation with LPS, which contributes to non-canonical inflammasome activation (Kayagaki et al., 2011), while the roles of CA in non-canonical inflammasome activation remain incompletely understood.

NLRP3 inflammasome activation is triggered by extracellular ATP existence, intracellular excessive ROS production, potassium efflux, calcium unbalance, and lysosome disruption (Meyers and Zhu, 2020). It was well known that abnormal ROS production may cause oxidative stress and subsequent tissue damage (Mullen et al., 2020). Numerous studies have reported that LPS administration increased the levels of ROS and reactive nitrogen species (RNS), which lead to NLRP3 inflammasome activation (Banoth and Cassel, 2018). Toll-like receptor (TLR) 4 activated by LPS drives tumor necrosis factor receptor-associated factor 6 (TRAF6) to translocate to mitochondria, resulting in enhancement of mitochondrial ROS production (Carneiro et al., 2018). CA is an important active isoflavone compound from Radix Astragali with a phenolic hydroxyl group. The phenolic hydroxyl groups on isoflavone can react with free radicals to terminate the chain reaction of free radicals. CA exhibits the protective effect through attenuating oxidative stress in a variety of diseases, including cardiotoxicity (Zhai et al., 2020), Alzheimer’s disease (Song et al., 2017), diabetes (Wang and Zhao, 2016), and ischemia-reperfusion injury (Ren et al., 2016). Consistent with these reports, our results showed that CA significantly attenuated oxidative stress in lung tissue and reduced mitochondrial ROS levels markedly in BMDMs. However, our results showed that CA did not alter NLRP3 and ASC expression, indicating that CA with phenolic hydroxyl group did not inhibit inflammasome activation through changing gene expression. Meanwhile, many studies have reported that CA downregulates NF-κB activity (Yang J. et al., 2019; Zhang et al., 2019). It is predictable that CA not only scavenges ROS but also inhibits NF-kB activity. It is well known that ROS increases NF-κB activity (Sun, 2017). One possible mechanism is that CA decreases NF-κB activity by reducing the level of ROS, which in turn inhibits IL-1β expression. However, few reports have shown the effect of CA on the relationship between ROS and inflammasome activation. Here, we used the mitochondria-targeted antioxidant to prove that mitochondrial ROS attenuated by CA contributes to inflammasome inactivation in macrophages, suggesting that mitochondria probably serve as a target for the treatment of ALI by CA.

In summary, this study has revealed the potential protective effect of CA as an anti-inflammatory candidate to counteract the inflammatory state *in vivo* and *in vitro*. Our data strongly suggest that the advantage of CA in the treatment of sepsis is the reversal of lung injury and the inhibition of excessive inflammasome activation. Moreover, the unique role of CA in reducing mitochondrial ROS in macrophages offers the possibility of a new therapeutic strategy for the treatment of sepsis. Further studies in clinical trials may provide more insight into the functional roles of CA in the treatment of ALI.

## Data Availability

The raw data supporting the conclusion of this article will be made available by the authors without undue reservation.
